# First detection of the adventive large rove beetle *Ocypus nitens* (Schrank) in Canada and an update of its Nearctic distribution using data generated by the public

**DOI:** 10.3897/BDJ.4.e11012

**Published:** 2016-11-16

**Authors:** Adam J. Brunke

**Affiliations:** ‡ Canadian National Collection of Insects, Arachnids and Nematodes, Ottawa, Canada

**Keywords:** faunistics, Staphylininae, Staphylinidae, Coleoptera, exotic, new record, Nearctic

## Abstract

The adventive rove beetle *Ocypus nitens* (Staphylinidae: Staphylininae) is newly recorded in Canada (Ontario) and the state of Vermont, and additional range expansion is documented. The updated distribution of this large, conspicuous species is based mostly on data from digital photographs posted by users of the online community BugGuide. All available data are summarized and made available as a DarwinCore dataset, and an updated distribution map is provided. Citizen-generated distributional data continues to be a valuable ally in the detection of adventive insects and the study of their distributional dynamics.

## Introduction

Hundreds of years of trade between the Nearctic and Palaeartic regions has resulted in the accidental introduction and successful establishment of many Central European beetles in North America. This is especially true for the rove beetles (Coleoptera: Staphylindae), where adventive species form about nine percent of the known Canadian fauna (Bousquet et al. 2013). Since the baseline information for Canada was published by Bousquet et al. (2013), additional species have been detected in North America with no sign of a decrease in introductions over time (Klimaszewski et al. 2013). Recently the large rove beetles in the subtribe Staphylinina (Staphylininae) occurring in northeastern North America were reviewed by Brunke et al. (2011) and a key was provided for their identification. The adventive species *Ocypus nitens* (Schrank) was reported by these authors from the eastern United States in Maine, Massachusetts, New Hampshire, New York and Rhode Island.

Only 5 years later, the known distribution of this conspicuous and relatively large species is increased to include Ontario, Canada, and the state of Vermont based mostly on photo-based occurrence data submitted to BugGuide, a North American digital insect collection created and curated by an online community of naturalists, insect enthusiasts and entomologists.

## Materials and methods

### Data

A single pinned specimen of *Ocypus nitens* from Ontario, Canada (A. B. T. Smith, Canadian Museum of Nature) was examined and identified by the author. Additional distributional data was sought from BugGuide by curating unidentified and identified photos placed to Staphylinidae, Staphylininae, Staphylinini or Staphylinina by other users. All photos previously identified as *O. nitens* were verified by the author. A complete DarwinCore compliant dataset of the new distribution data (1 pinned specimen and 26 photographic records) was compiled by the author. For photo-based records, Bugguide photo identifiers were entered for 'catalognumber' in the Darwin Core schema, photographers were entered under 'recordedBy' and a resolveable URL to the photo record is provided under 'source'. Additional databases were searched (Flickr, Barcode of Life) for North American records but no further data could be added.

### Figures

The distribution map was created with ArcMap and further modified using Adobe Illustrator (CS5).

## Taxon treatments

### Ocypus nitens

(Schrank 1781)

Ocypus nitens For synonymy and literature list see Schülke and Smetana (2015)

#### Materials

- **Type status:** Other material. **Occurrence:** catalogNumber: AJB0000369; recordedBy: A.B.T. Smith; individualCount: 1; lifeStage: adult; **Taxon:** scientificName: Ocypus nitens; family: Staphylinidae; genus: Ocypus; specificEpithet: Ocypusnitens; scientificNameAuthorship: (Schrank, 1781); **Location:** country: Canada; stateProvince: Ontario; county: Peel; verbatimLocality: 13 km NE Orangeville, Glen-Haffy Rd.; decimalLatitude: 43.9386; decimalLongitude: -79.9364; georeferenceProtocol: label; georeferenceSources: GPS; **Identification:** identifiedBy: A. J. Brunke; dateIdentified: 2016; **Event:** samplingProtocol: none specified; eventDate: 25.V.2014; habitat: Maple-Beech forest; **Record Level:** type: Other material; language: en; institutionID: Canadian Museum of Nature; institutionCode: CMNC; basisOfRecord: PreservedSpecimen
- **Type status:** Other material. **Occurrence:** catalogNumber: 1045283; recordedBy: "sh11"; individualCount: 1; lifeStage: adult; otherCatalogNumbers: 1045284; **Taxon:** scientificName: Ocypus nitens; family: Staphylinidae; genus: Ocypus; specificEpithet: Ocypusnitens; scientificNameAuthorship: (Schrank, 1781); **Location:** country: Canada; stateProvince: Ontario; county: Peel; verbatimLocality: Mississauga; decimalLatitude: 43.66; decimalLongitude: -79.64; georeferenceProtocol: GPS; georeferenceSources: Google Earth; **Identification:** identifiedBy: A. J. Brunke; dateIdentified: 2016; **Event:** samplingProtocol: none specified; eventDate: 13.VI.2014; **Record Level:** type: Other material; language: en; institutionID: Bugguide.net; basisOfRecord: Photograph; source: http://bugguide.net/node/view/1045283
- **Type status:** Other material. **Occurrence:** catalogNumber: 1005679; recordedBy: P. Hollinger; individualCount: 1; lifeStage: adult; **Taxon:** scientificName: Ocypus nitens; family: Staphylinidae; genus: Ocypus; specificEpithet: Ocypusnitens; scientificNameAuthorship: (Schrank, 1781); **Location:** country: United States; stateProvince: Vermont; county: Windsor; verbatimLocality: Sharon; decimalLatitude: 43.79; decimalLongitude: -72.45; georeferenceProtocol: GPS; georeferenceSources: Google Earth; **Identification:** identifiedBy: A. J. Brunke; dateIdentified: 2016; **Event:** samplingProtocol: none specified; eventDate: 27.IX.2014; habitat: side of house; **Record Level:** type: Other material; language: en; institutionID: Bugguide.net; basisOfRecord: Photograph; source: http://bugguide.net/node/view/1005679
- **Type status:** Other material. **Occurrence:** catalogNumber: 773583; recordedBy: F. Kynd; individualCount: 1; lifeStage: adult; **Taxon:** scientificName: Ocypus nitens; family: Staphylinidae; genus: Ocypus; specificEpithet: Ocypusnitens; scientificNameAuthorship: (Schrank, 1781); **Location:** country: United States; stateProvince: Maine; county: Waldo; verbatimLocality: Searsmont; decimalLatitude: 44.36; decimalLongitude: -69.2; georeferenceProtocol: GPS; georeferenceSources: Google Earth; **Identification:** identifiedBy: A. J. Brunke; dateIdentified: 2016; **Event:** samplingProtocol: none specified; eventDate: 17.V.2013; **Record Level:** type: Other material; language: en; institutionID: Bugguide.net; basisOfRecord: Photograph; source: http://bugguide.net/node/view/773583
- **Type status:** Other material. **Occurrence:** catalogNumber: 721822; recordedBy: S. Nanz; individualCount: 1; lifeStage: adult; otherCatalogNumbers: 722232; **Taxon:** scientificName: Ocypus nitens; family: Staphylinidae; genus: Ocypus; specificEpithet: Ocypusnitens; scientificNameAuthorship: (Schrank, 1781); **Location:** country: United States; stateProvince: Maine; county: Knox; verbatimLocality: Vinalhaven; decimalLatitude: 44.05; decimalLongitude: -68.83; georeferenceProtocol: GPS; georeferenceSources: Google Earth; **Identification:** identifiedBy: A. J. Brunke; dateIdentified: 2016; **Event:** samplingProtocol: none specified; eventDate: 15.VIII.2011; **Record Level:** type: Other material; language: en; institutionID: Bugguide.net; basisOfRecord: Photograph; source: http://bugguide.net/node/view/721822
- **Type status:** Other material. **Occurrence:** catalogNumber: 1176943; recordedBy: B. Woo; individualCount: 1; lifeStage: adult; **Taxon:** scientificName: Ocypus nitens; family: Staphylinidae; genus: Ocypus; specificEpithet: Ocypusnitens; scientificNameAuthorship: (Schrank, 1781); **Location:** country: United States; stateProvince: Maine; county: York; verbatimLocality: Kennebunk Dump; decimalLatitude: 43.39; decimalLongitude: -70.59; georeferenceProtocol: GPS; georeferenceSources: Google Earth; **Identification:** identifiedBy: A. J. Brunke; dateIdentified: 2016; **Event:** samplingProtocol: none specified; eventDate: 25.XII.2015; habitat: under rock at field edge; **Record Level:** type: Other material; language: en; institutionID: Bugguide.net; basisOfRecord: Photograph; source: http://bugguide.net/node/view/1176943
- **Type status:** Other material. **Occurrence:** catalogNumber: 589731; recordedBy: B. Woo; individualCount: 1; lifeStage: adult; otherCatalogNumbers: 589732; **Taxon:** scientificName: Ocypus nitens; family: Staphylinidae; genus: Ocypus; specificEpithet: Ocypusnitens; scientificNameAuthorship: (Schrank, 1781); **Location:** country: United States; stateProvince: Maine; county: York; verbatimLocality: Parson's beach, Kennebunk; decimalLatitude: 43.35; decimalLongitude: -70.52; georeferenceProtocol: GPS; georeferenceSources: Google Earth; **Identification:** identifiedBy: A. J. Brunke; dateIdentified: 2016; **Event:** samplingProtocol: none specified; eventDate: 23.X.2011; habitat: ocean beach under driftwood; **Record Level:** type: Other material; language: en; institutionID: Bugguide.net; basisOfRecord: Photograph; source: http://bugguide.net/node/view/589731
- **Type status:** Other material. **Occurrence:** catalogNumber: 501820; recordedBy: B. Woo; individualCount: 1; lifeStage: adult; otherCatalogNumbers: 501821; **Taxon:** scientificName: Ocypus nitens; family: Staphylinidae; genus: Ocypus; specificEpithet: Ocypusnitens; scientificNameAuthorship: (Schrank, 1781); **Location:** country: United States; stateProvince: Maine; county: York; verbatimLocality: Kennebunk; decimalLatitude: 43.39; decimalLongitude: -70.59; georeferenceProtocol: GPS; georeferenceSources: Google Earth; **Identification:** identifiedBy: A. J. Brunke; dateIdentified: 2016; **Event:** samplingProtocol: none specified; eventDate: 30.III.2011; habitat: leaf litter; **Record Level:** type: Other material; language: en; institutionID: Bugguide.net; basisOfRecord: Photograph; source: http://bugguide.net/node/view/501820
- **Type status:** Other material. **Occurrence:** catalogNumber: 862165; recordedBy: P. Crocket; individualCount: 1; lifeStage: adult; **Taxon:** scientificName: Ocypus nitens; family: Staphylinidae; genus: Ocypus; specificEpithet: Ocypusnitens; scientificNameAuthorship: (Schrank, 1781); **Location:** country: United States; stateProvince: New York; county: Onondaga; verbatimLocality: Fabius; decimalLatitude: 42.84; decimalLongitude: -75.99; georeferenceProtocol: GPS; georeferenceSources: Google Earth; **Identification:** identifiedBy: A. J. Brunke; dateIdentified: 2016; **Event:** samplingProtocol: none specified; eventDate: 3.XI.2013; habitat: on country road; **Record Level:** type: Other material; language: en; institutionID: Bugguide.net; basisOfRecord: Photograph; source: http://bugguide.net/node/view/862165
- **Type status:** Other material. **Occurrence:** catalogNumber: 761929; recordedBy: S. Ausubel; individualCount: 1; lifeStage: adult; otherCatalogNumbers: 761930; **Taxon:** scientificName: Ocypus nitens; family: Staphylinidae; genus: Ocypus; specificEpithet: Ocypusnitens; scientificNameAuthorship: (Schrank, 1781); **Location:** country: United States; stateProvince: New York; county: Dutchess; verbatimLocality: James Baird State Park; decimalLatitude: 41.69; decimalLongitude: -73.79; georeferenceProtocol: GPS; georeferenceSources: Google Earth; **Identification:** identifiedBy: A. J. Brunke; dateIdentified: 2016; **Event:** samplingProtocol: none specified; eventDate: 20.IV.2013; **Record Level:** type: Other material; language: en; institutionID: Bugguide.net; basisOfRecord: Photograph; source: http://bugguide.net/node/view/761929
- **Type status:** Other material. **Occurrence:** catalogNumber: 504056; recordedBy: K. Hillig; individualCount: 1; lifeStage: adult; otherCatalogNumbers: 504296, 504297, 504300; **Taxon:** scientificName: Ocypus nitens; family: Staphylinidae; genus: Ocypus; specificEpithet: Ocypusnitens; scientificNameAuthorship: (Schrank, 1781); **Location:** country: United States; stateProvince: New York; county: Saratoga; verbatimLocality: Ballston Lake; decimalLatitude: 42.95; decimalLongitude: -73.85; georeferenceProtocol: GPS; georeferenceSources: Google Earth; **Identification:** identifiedBy: A. J. Brunke; dateIdentified: 2016; **Event:** samplingProtocol: none specified; eventDate: 8.IV.2011; habitat: under loose bark on white pine log; **Record Level:** type: Other material; language: en; institutionID: Bugguide.net; basisOfRecord: Photograph; source: http://bugguide.net/node/view/504056
- **Type status:** Other material. **Occurrence:** catalogNumber: 387557; recordedBy: K. Hillig; individualCount: 1; lifeStage: adult; **Taxon:** scientificName: Ocypus nitens; family: Staphylinidae; genus: Ocypus; specificEpithet: Ocypusnitens; scientificNameAuthorship: (Schrank, 1781); **Location:** country: United States; stateProvince: New York; county: Saratoga; verbatimLocality: Ballston Lake; decimalLatitude: 42.95; decimalLongitude: -73.85; georeferenceProtocol: GPS; georeferenceSources: Google Earth; **Identification:** identifiedBy: A. J. Brunke; dateIdentified: 2016; **Event:** samplingProtocol: none specified; eventDate: 21.IV.2010; habitat: under porch light; **Record Level:** type: Other material; language: en; institutionID: Bugguide.net; basisOfRecord: Photograph; source: http://bugguide.net/node/view/387557
- **Type status:** Other material. **Occurrence:** catalogNumber: 158490; recordedBy: D. Thombs; individualCount: 2; lifeStage: adult; **Taxon:** scientificName: Ocypus nitens; family: Staphylinidae; genus: Ocypus; specificEpithet: Ocypusnitens; scientificNameAuthorship: (Schrank, 1781); **Location:** country: United States; stateProvince: Rhode Island; county: Newport; verbatimLocality: Tiverton; decimalLatitude: 41.63; decimalLongitude: -71.21; georeferenceProtocol: GPS; georeferenceSources: Google Earth; **Identification:** identifiedBy: A. J. Brunke; dateIdentified: 2016; **Event:** samplingProtocol: none specified; eventDate: 13.X.2007; **Record Level:** type: Other material; language: en; institutionID: Bugguide.net; basisOfRecord: Photograph; source: http://bugguide.net/node/view/158490
- **Type status:** Other material. **Occurrence:** catalogNumber: 788532; recordedBy: M. Jacobson; individualCount: 1; lifeStage: adult; **Taxon:** scientificName: Ocypus nitens; family: Staphylinidae; genus: Ocypus; specificEpithet: Ocypusnitens; scientificNameAuthorship: (Schrank, 1781); **Location:** country: United States; stateProvince: New Hampshire; county: Hillsborough; verbatimLocality: Hollis; decimalLatitude: 42.74; decimalLongitude: -71.59; georeferenceProtocol: GPS; georeferenceSources: Google Earth; **Identification:** identifiedBy: A. J. Brunke; dateIdentified: 2016; **Event:** samplingProtocol: none specified; eventDate: 27.IV.2012; **Record Level:** type: Other material; language: en; institutionID: Bugguide.net; basisOfRecord: Photograph; source: http://bugguide.net/node/view/788532
- **Type status:** Other material. **Occurrence:** catalogNumber: 131220; recordedBy: T. Murray; individualCount: 1; lifeStage: adult; **Taxon:** scientificName: Ocypus nitens; family: Staphylinidae; genus: Ocypus; specificEpithet: Ocypusnitens; scientificNameAuthorship: (Schrank, 1781); **Location:** country: United States; stateProvince: New Hampshire; county: Hillsborough; verbatimLocality: Antrim; decimalLatitude: 43.03; decimalLongitude: -71.94; georeferenceProtocol: GPS; georeferenceSources: Google Earth; **Identification:** identifiedBy: A. J. Brunke; dateIdentified: 2016; **Event:** samplingProtocol: none specified; eventDate: 25.VII.2007; **Record Level:** type: Other material; language: en; institutionID: Bugguide.net; basisOfRecord: Photograph; source: http://bugguide.net/node/view/131220
- **Type status:** Other material. **Occurrence:** catalogNumber: 104927; recordedBy: S. Price; individualCount: 1; lifeStage: adult; **Taxon:** scientificName: Ocypus nitens; family: Staphylinidae; genus: Ocypus; specificEpithet: Ocypusnitens; scientificNameAuthorship: (Schrank, 1781); **Location:** country: United States; stateProvince: Massachusetts; county: Worchester; verbatimLocality: Fitchburg; decimalLatitude: 42.58; decimalLongitude: -71.8; georeferenceProtocol: GPS; georeferenceSources: Google Earth; **Identification:** identifiedBy: A. J. Brunke; dateIdentified: 2016; **Event:** samplingProtocol: none specified; eventDate: 22.IV.2007; habitat: inside house; **Record Level:** type: Other material; language: en; institutionID: Bugguide.net; basisOfRecord: Photograph; source: http://bugguide.net/node/view/104927
- **Type status:** Other material. **Occurrence:** catalogNumber: 624308; recordedBy: Marc DiGirolomo; individualCount: 1; lifeStage: adult; **Taxon:** scientificName: Ocypus nitens; family: Staphylinidae; genus: Ocypus; specificEpithet: Ocypusnitens; scientificNameAuthorship: (Schrank, 1781); **Location:** country: United States; stateProvince: Massachusetts; county: Worchester; verbatimLocality: Holden; decimalLatitude: 42.35; decimalLongitude: -71.87; georeferenceProtocol: GPS; georeferenceSources: Google Earth; **Identification:** identifiedBy: A. J. Brunke; dateIdentified: 2016; **Event:** samplingProtocol: none specified; eventDate: 2.IV.2010; **Record Level:** type: Other material; language: en; institutionID: Bugguide.net; basisOfRecord: Photograph; source: http://bugguide.net/node/view/624308
- **Type status:** Other material. **Occurrence:** catalogNumber: 1158654; recordedBy: J. Crockwell; individualCount: 1; lifeStage: adult; **Taxon:** scientificName: Ocypus nitens; family: Staphylinidae; genus: Ocypus; specificEpithet: Ocypusnitens; scientificNameAuthorship: (Schrank, 1781); **Location:** country: United States; stateProvince: Massachusetts; county: Berkshire; verbatimLocality: Old Mill Trail, Hinsdale; decimalLatitude: 42.44; decimalLongitude: -73.13; georeferenceProtocol: GPS; georeferenceSources: Google Earth; **Identification:** identifiedBy: A. J. Brunke; dateIdentified: 2016; **Event:** samplingProtocol: none specified; eventDate: 20.X.2015; habitat: forest trail, after sunset; **Record Level:** type: Other material; language: en; institutionID: Bugguide.net; basisOfRecord: Photograph; source: http://bugguide.net/node/view/1158654
- **Type status:** Other material. **Occurrence:** catalogNumber: 1026658; recordedBy: J. Crockwell; individualCount: 1; lifeStage: adult; **Taxon:** scientificName: Ocypus nitens; family: Staphylinidae; genus: Ocypus; specificEpithet: Ocypusnitens; scientificNameAuthorship: (Schrank, 1781); **Location:** country: United States; stateProvince: Massachusetts; county: Berkshire; verbatimLocality: Ashuwillticook Trail, Lanesborough; decimalLatitude: 42.52; decimalLongitude: -73.23; georeferenceProtocol: GPS; georeferenceSources: Google Earth; **Identification:** identifiedBy: A. J. Brunke; dateIdentified: 2016; **Event:** samplingProtocol: none specified; eventDate: 24.XI.2014; **Record Level:** type: Other material; language: en; institutionID: Bugguide.net; basisOfRecord: Photograph; source: http://bugguide.net/node/view/1026658
- **Type status:** Other material. **Occurrence:** catalogNumber: 645048; recordedBy: B. Z.; individualCount: 1; lifeStage: adult; **Taxon:** scientificName: Ocypus nitens; family: Staphylinidae; genus: Ocypus; specificEpithet: Ocypusnitens; scientificNameAuthorship: (Schrank, 1781); **Location:** country: United States; stateProvince: Massachusetts; county: Berkshire; verbatimLocality: Williamstown; decimalLatitude: 42.71; decimalLongitude: -73.21; georeferenceProtocol: GPS; georeferenceSources: Google Earth; **Identification:** identifiedBy: A. J. Brunke; dateIdentified: 2016; **Event:** samplingProtocol: none specified; eventDate: 13.V.2012; **Record Level:** type: Other material; language: en; institutionID: Bugguide.net; basisOfRecord: Photograph; source: http://bugguide.net/node/view/645048
- **Type status:** Other material. **Occurrence:** catalogNumber: 630438; recordedBy: B. Z.; individualCount: 1; lifeStage: adult; **Taxon:** scientificName: Ocypus nitens; family: Staphylinidae; genus: Ocypus; specificEpithet: Ocypusnitens; scientificNameAuthorship: (Schrank, 1781); **Location:** country: United States; stateProvince: Massachusetts; county: Essex; verbatimLocality: Newbury; decimalLatitude: 42.77; decimalLongitude: -70.84; georeferenceProtocol: GPS; georeferenceSources: Google Earth; **Identification:** identifiedBy: A. J. Brunke; dateIdentified: 2016; **Event:** samplingProtocol: none specified; eventDate: 15.IV.2012; **Record Level:** type: Other material; language: en; institutionID: Bugguide.net; basisOfRecord: Photograph; source: http://bugguide.net/node/view/630438
- **Type status:** Other material. **Occurrence:** catalogNumber: 97964; recordedBy: T. Murray; individualCount: 1; lifeStage: adult; **Taxon:** scientificName: Ocypus nitens; family: Staphylinidae; genus: Ocypus; specificEpithet: Ocypusnitens; scientificNameAuthorship: (Schrank, 1781); **Location:** country: United States; stateProvince: Massachusetts; county: Essex; verbatimLocality: North Andover; decimalLatitude: 42.7; decimalLongitude: -71.13; georeferenceProtocol: GPS; georeferenceSources: Google Earth; **Identification:** identifiedBy: A. J. Brunke; dateIdentified: 2016; **Event:** samplingProtocol: none specified; eventDate: 11.III.2007; **Record Level:** type: Other material; language: en; institutionID: Bugguide.net; basisOfRecord: Photograph; source: http://bugguide.net/node/view/97964
- **Type status:** Other material. **Occurrence:** catalogNumber: 1058649; recordedBy: "ophis"; individualCount: 1; lifeStage: adult; **Taxon:** scientificName: Ocypus nitens; family: Staphylinidae; genus: Ocypus; specificEpithet: Ocypusnitens; scientificNameAuthorship: (Schrank, 1781); **Location:** country: United States; stateProvince: Massachusetts; county: Norfolk; verbatimLocality: Blue Hills Reservation, Randolph; decimalLatitude: 42.21; decimalLongitude: -71.07; georeferenceProtocol: GPS; georeferenceSources: Google Earth; **Identification:** identifiedBy: A. J. Brunke; dateIdentified: 2016; **Event:** samplingProtocol: none specified; eventDate: 18.IV.2015; habitat: punky wood, log in oak/pine woods; **Record Level:** type: Other material; language: en; institutionID: Bugguide.net; basisOfRecord: Photograph; source: http://bugguide.net/node/view/1058649
- **Type status:** Other material. **Occurrence:** catalogNumber: 384163; recordedBy: T. Murray; individualCount: 1; lifeStage: adult; **Taxon:** scientificName: Ocypus nitens; family: Staphylinidae; genus: Ocypus; specificEpithet: Ocypusnitens; scientificNameAuthorship: (Schrank, 1781); **Location:** country: United States; stateProvince: Massachusetts; county: Norfolk; verbatimLocality: Sharon; decimalLatitude: 42.12; decimalLongitude: -71.18; georeferenceProtocol: GPS; georeferenceSources: Google Earth; **Identification:** identifiedBy: A. J. Brunke; dateIdentified: 2016; **Event:** samplingProtocol: none specified; eventDate: 10.IV.2010; **Record Level:** type: Other material; language: en; institutionID: Bugguide.net; basisOfRecord: Photograph; source: http://bugguide.net/node/view/384163
- **Type status:** Other material. **Occurrence:** catalogNumber: 357461; recordedBy: C. Eiseman; individualCount: 1; lifeStage: adult; **Taxon:** scientificName: Ocypus nitens; family: Staphylinidae; genus: Ocypus; specificEpithet: Ocypusnitens; scientificNameAuthorship: (Schrank, 1781); **Location:** country: United States; stateProvince: Massachusetts; county: Hampshire; verbatimLocality: Pelham; decimalLatitude: 42.39; decimalLongitude: -72.4; georeferenceProtocol: GPS; georeferenceSources: Google Earth; **Identification:** identifiedBy: A. J. Brunke; dateIdentified: 2016; **Event:** samplingProtocol: none specified; eventDate: 8.XII.2009; habitat: kitchen floor; **Record Level:** type: Other material; language: en; institutionID: Bugguide.net; basisOfRecord: Photograph; source: http://bugguide.net/node/view/357461
- **Type status:** Other material. **Occurrence:** catalogNumber: 8435; recordedBy: T. DiTerlizzi; individualCount: 1; lifeStage: adult; **Taxon:** scientificName: Ocypus nitens; family: Staphylinidae; genus: Ocypus; specificEpithet: Ocypusnitens; scientificNameAuthorship: (Schrank, 1781); **Location:** country: United States; stateProvince: Massachusetts; county: Hampshire; verbatimLocality: Amherst; decimalLatitude: 42.38; decimalLongitude: -72.52; georeferenceProtocol: GPS; georeferenceSources: Google Earth; **Identification:** identifiedBy: A. J. Brunke; dateIdentified: 2016; **Event:** samplingProtocol: none specified; eventDate: 1.XI.2004; habitat: garage; **Record Level:** type: Other material; language: en; institutionID: Bugguide.net; basisOfRecord: Photograph; source: http://bugguide.net/node/view/8435
- **Type status:** Other material. **Occurrence:** catalogNumber: 1217183; recordedBy: A. Hunt; individualCount: 1; lifeStage: adult; otherCatalogNumbers: 1217184, 1217185, 1217188; **Taxon:** scientificName: Ocypus nitens; family: Staphylinidae; genus: Ocypus; specificEpithet: Ocypusnitens; scientificNameAuthorship: (Schrank, 1781); **Location:** country: United States; stateProvince: Rhode Island; county: Washington; verbatimLocality: Block Island; decimalLatitude: 41.17; decimalLongitude: -71.57; georeferenceProtocol: GPS; georeferenceSources: Google Earth; **Identification:** identifiedBy: A. J. Brunke; dateIdentified: 2016; **Event:** samplingProtocol: none specified; eventDate: 30.IV.2016; habitat: dirt path near pond; **Record Level:** type: Other material; language: en; institutionID: Bugguide.net; basisOfRecord: Photograph; source: http://bugguide.net/node/view/1217183

#### Diagnosis

**Figure 1.**
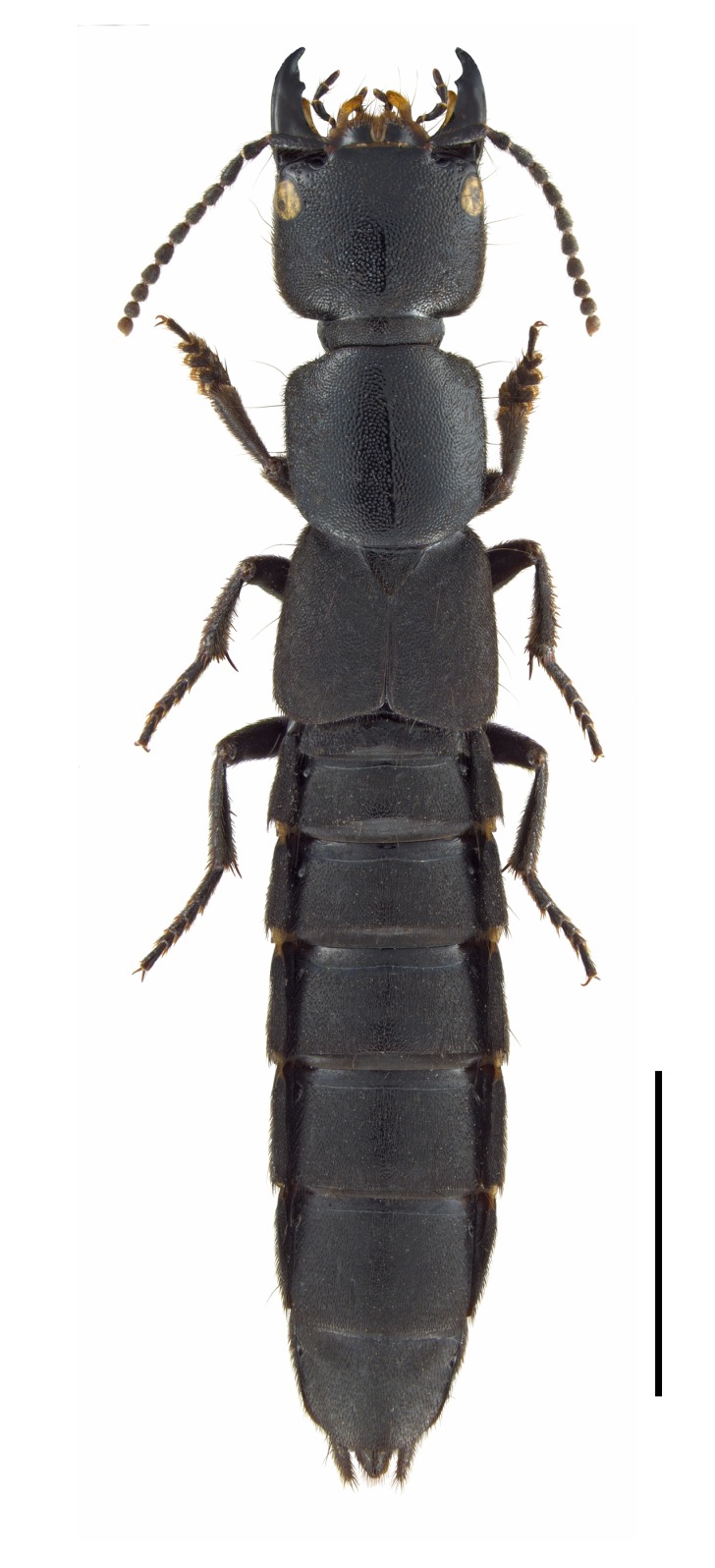
*Ocypus nitens* (Schrank), dorsal habitus. Photograph by Harald Schillhammer, reproduced with permission.

In the eastern North American fauna, *O. nitens* can be recognized using the diagnosis given by Brunke et al. (2011)(http://cjai.biologicalsurvey.ca/bnkmm_12/species_pages/ocyn.html) but can also be reliably identified from photos by its characteristically convex head and pronotum, and the relatively short elytra compared to the pronotum (Fig. 1). Superficially similar species of *Tasgius* (http://bugguide.net/node/view/868738), also adventive in North America, are more flattened dorsoventrally compared to *Ocypus nitens* (http://bugguide.net/node/view/862165).

#### Distribution

**Figure 2.**
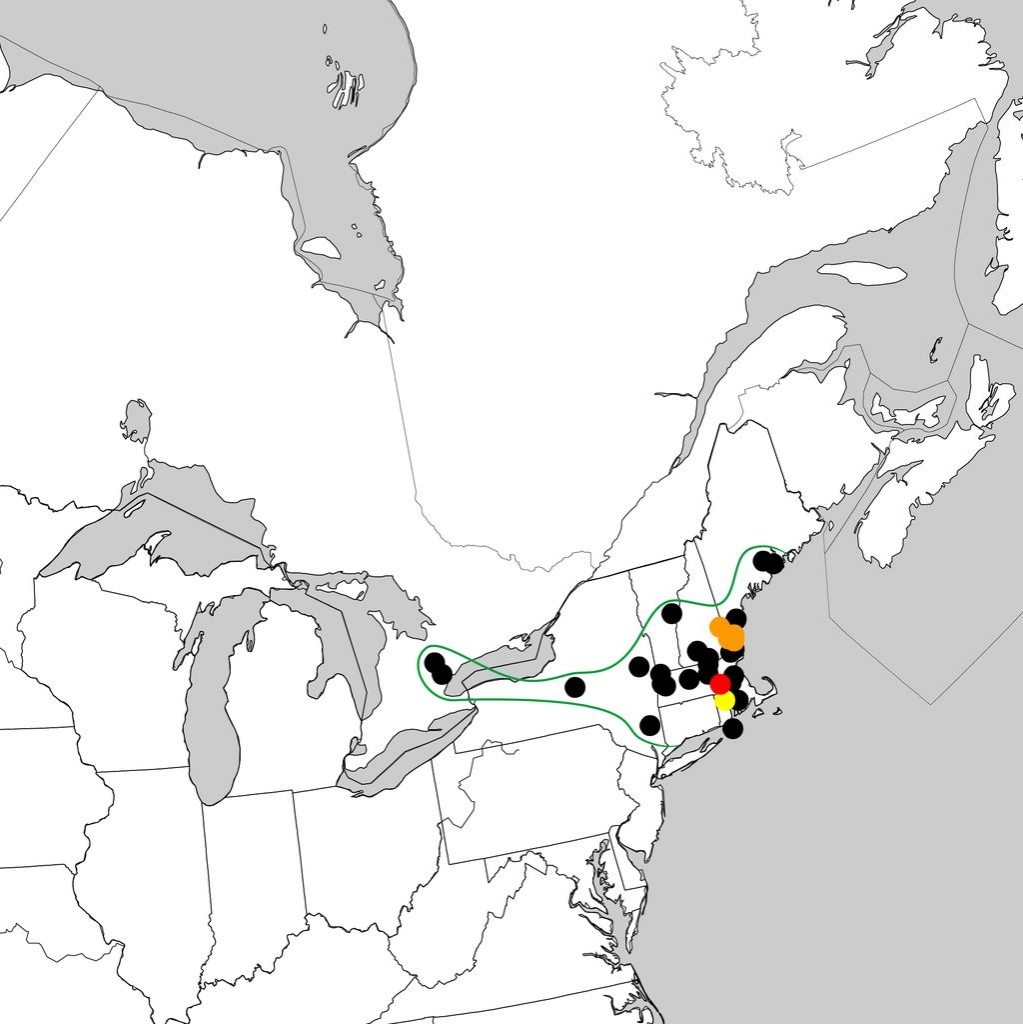
Nearctic distribution of *Ocypus nitens* (Schrank). Red circle indicates oldest record in 1944; orange circles indicate records 1980-1989; yellow circles indicate records 1990-1999; black circles indicate records 2000-present. Data includes those that were presented by Newton (1987) and Brunke et al. (2011).

North America (adventive) (Fig. 2​): Canada (ON); United States (MA, ME, NH, NY, RI, VT).

*Ocypus nitens* is newly recorded from Canada (Ontario) and the state of Vermont (United States). Its native range in the Palaeartic Region includes Europe, Russia, Iran and Turkey (Schülke and Smetana 2015).

#### Taxon discussion

For a period of more than fifty years (1944-2000), the adventive species *Ocypus nitens* was known only from a small area in New England (Newton 1987, Brunke et al. 2011; Fig. 2 (colored dots)). The expansion of its range westward (Ontario and New York) and northward (Maine) appears to have been recent and rapid, though the reasons for this remain unknown. Newton (1987) reported non-brachypterous individuals in North America, which may be expected to fly, at least occasionally. Their presence on several islands in New England supports this, though they could have been transported there with soil or other organic debris. There is a possibility that *Ocypus nitens* remained undetected in Ontario and New York for much of this fifty year period due to a lack of sampling. However, this scenario is considered unlikely, especially for southern Ontario, which has had a long history of collecting by professionals and students in rural and urban habitats. Furthermore, many adventive staphylinid species that now occur broadly in eastern North America were first detected in southern Canada during this period (Klimaszewski et al. 2013). *Ocypus nitens* is large (12-20 mm, (Schillhammer 2012)) and typically found in human disturbed habitats including woodlots and backyards, making it likely to be encountered by homeowners, naturalists and entomologists alike. Indeed, this is reflected in the number of recent photographic records available on BugGuide. This online, community-based resource continues to be an important source of first detections of adventive species in Canada, the United States, or the entire Western Hemisphere (Kaldari et al. 2011, Carlson et al. 2012). Gibbs and Sheffield (2009) demonstrated that a rapid range expansion of an adventive bee species was captured by digital insect collections, including BugGuide, and emphasized their effectiveness when coupled with adequate identification resources or taxonomic expertise. Citizen science-based observation data, especially when verified, have become a cost and time-effective option to answer large-scale questions about insect distributions for certain groups that are taxonomically well-known and conspicuous, such as lady beetles (Coccinellidae) (Gardiner et al. 2012).

*Ocypus nitens* joins *Ocypus aeneocephalus* (DeGeer), *Tasgius ater* (Gravenhorst), *T. melanarius* (Heer) and *T. winkleri* (Bernhauer) as the largest adventive staphylinids established in Canada and can be expected to eventually occur in Quebec and the Maritime Provinces. The even larger adventive species *Ocypus olens* (Müller) has become established in western North America but is still restricted to the western United States (Washington, Oregon, California and Arizona) and unknown from Canada (Newton et al. 2001, BugGuide). This is another staphylinid that would be easily monitored by citizen science. The impact of these large species on related, native edaphic rove beetles (*Platydracus* and *Dinothenarus*) occupying similar microhabitats is unknown but several *Platydracus* species appear to be less abundant than they were at the beginning of the 20th century (Brunke et al. 2011).

## Supplementary Material

XML Treatment for Ocypus nitens
